# Protective Effect of *Urtica dioica* L. (Urticaceae) on Morphometric and Morphologic Alterations of Seminiferous Tubules in STZ Diabetic Rats

**Published:** 2011

**Authors:** Mohammad Jafar Golalipour, Babak Kabiri Balajadeh, Soraya Ghafari, Ramin Azarhosh, Vahid Khori

**Affiliations:** 1 *Department of Anatomy (Embryology and Histology), Golestan University of Medical Sciences, Gorgan, Iran*; 2 *Department of Pathology, Golestan University of Medical Sciences, Gorgan, Iran*; 3 *Department of Pharmacology, Golestan University of Medical Sciences, Gorgan, Iran*

**Keywords:** Diabet mellitus, Morphometry, Rats, Seminiferous tubules, Testis, Urtica dioica

## Abstract

**Objective(s):**

*Urtica dioica *L. has been known as a medicinal plant in the world. This study was conducted to determine the effects of the hydroalcoholic extract of *Urtica dioica *leaves on seminiferous tubules of diabetic rats.

**Materials and Methods:**

Animals were allocated to control, diabetic and protective groups. Treated animals received extract of *U. dioica *(100 mg/ kg/ day) IP for the first 5 days and STZ injection on the 6th day. After 5 weeks, testes removed and stained with H&E technique.

**Results:**

Tubular cell disintegration, sertoli and spermatogonia cell vacuolization, and decrease in sperm concentration observed in diabetic in comparison with control and protective groups. External seminiferous tubular diameter and seminiferous epithelial height significantly reduced (*P*< 0.05) in diabetic compared with controls, and these parameters increased (*P*< 0.05) in the treated compared with diabetics.

**Conclusion:**

Hydroalcoholic extract of *U. dioica*, before induction of diabetes; has protective role on seminiferous tubules alterations.

## Introduction

Diabetes mellitus is a disease due to abnormality of carbohydrate metabolism and it is mainly linked to low blood insulin level or insensitivity of target organs to insulin (1). Sexual dysfunction is associated with diabetes in men and experimental animals (2). The effects of diabetes on the male reproductive organs include testicular alterations associated with defective spermatogenesis (3).

Histopathological and histomorphometric alterations in seminiferous tubules have been reported in streptozotocin – induced diabetic animals (4,5).

In recent years, there has been renewed interest in plant medicine for the treatment of different diseases (6). Isolated studies screened various plants having “folk medicine reputation” by biochemical test for this antidiabetogenic effect (7).

*Urtica dioica*, commonly known the stinging nettle, is a plant that grows in plenty in many countries namely Iran, Greece and Turkey. It has a long history of use in traditional medicine, as well as being used for food. The examples of its beneficial properties are as an analgesic, antihyperglycemic, antioxidant, antihyperlipidemic and an antithrombotic agent (8). 

The prophylactic effects of *U. dioica* in preventing from complications of diabetes on reproductive male system have not been shown clearly. Therefore, this study was performed to determine the protective effects of the hydroalcoholic extract of *U. dioica* leaves on histopathological and morphometrical alterations of seminiferous tubules in streptozotocin– induced diabetic rats. 

## Materials and Methods

This experimental study was conducted in the Faculty of Medicine, Golestan (Gorgan) . Approval for this study was acquired from the Animal Care and Ethics Committee of the Golestan University of Medical Sciences.

***Plant material***

*U. dioica* L. (Urticaceae) leaves were collected from cultivated plant, from suburb of Gorgan, northern () and taxonomically identified in the Department of Pharmacognosy, Mazandaran University of Medical Sciences. A voucher specimen (5-77-1) was deposited in the Herbarium of Mazandaran University.

***Preparation of extract of U. dioica***

The dried and powered *U. dioica* leaves (400 g) were percolated by Ethanol (45%) solvent. In brief, the dried leaf of *U. dioica* (by using hot air 35-40 ºC) was powdered by mechanical milling. Prelinering maceration during 5 hr was done and the product was percolated and mixed during 48 hours. The extract was filtrated (0.8 Micron) and spray dried in a lab plant SD4 spray drier (lab plant ltd, ). Spectrophotometric assay was carried out to determine the concentration of phenolic and flavonid content of *U. dioica* leaves extract. Also antioxidant activity was measured by DPPH method. 

***Animals***

Eighteen male Wistar rats (125-175 g) were used for this study. Ethical approval and animal care were in accordance with the principles of the regulations in use at Gorgan University of Medical Sciences, . The rats were housed in groups of three in standard animal cages and kept under standard laboratory conditions in Gorgan University of Medical Sciences. Animals had free access to rat pelleted chow and tap water.

***Experimental design***

The rats were divided into three groups. Each group included six animals.

Group I: control rats: Animals received intraperitoneal (IP) saline for six days. Group II: STZ-induced diabetic rats: Animals received IP saline for 5 days then challenged with 80 mg/ kg STZ IP on the 6th day. 

Group III: protective group, received IP injection of 100 mg/ kg (8) *U. dioica* leaves extract (dissolved in distal water) for 5 days then received one dose of 80 mg/kg STZ IP on the 6th day. 

The animals in three groups did not receive any matter between 6 and 35 days. The animals were sacrified at 35th day of experiment.

Hyperglycemia (blood glucose range of above 200 mg/dl) was induced with single i.p. injection of streptozotocin (STZ) with a dose of 80 mg/ kg body weight dissolved in distilled water just before overnight fasting. Glucose concentration was measured in the blood of the rats’ tail veins with an Accu-Check Active blood glucose monitor test strip.

***Glucose tolerance test***

Intraperitoneal glucose tolerance test (GTT) was performed on 16 hr fasted rats using 2 grams glucose/ kg-body weight. In all groups, blood was collected from the animals by tail snipping at 0, 30, 60 and 120 min after glucose load. Glucose tolerance test was performed at the beginning of study and on 20th and 35th days of experiment in control and diabetic groups. Also Glucose tolerance test was performed at the beginning of study, before injection of STZ on 6th day and after i.p. injection of STZ on 20th and 35th days of experiment in protective group.

***Tissue processing***

After five weeks from the beginning of the experiment, all animals in the three groups were deeply anesthetized with chloroform. After cervical dislocation, the left testis of each experimental rat was extracted and fixed in bouin’s fixative. Slices were fixed at four mm thickness and embedded in paraffin wax after overnight automatical processing. 

Ten sections of each specimen, taken from the left testis, hematoxylin and eosin stained sections (32) at four µm thickness with 300 µm distance, were used for morphometric analyses. The picture of each section was taken by the Olympus BX-51T-32E01 research microscope connected to DP12 Camera with 3.34 million pixel resolution and Olysia Bio software (from: Olympus Optical Co. LTD, Tokyo-Japan) under magnification of 100× and 400×. Twenty seminiferous tubules in stage VI–X were measured in each section. 

***Morphometric study***

A morphometric study for each chosen seminiferous tubule that included external seminiferous tubular diameter (STD) and seminiferous epithelial height (SEH) were measured by Olysia Bio software. 

***Statistical analysis***

General linear model and repeated measures were used to analyze the data of glucose. Comparison of morphometric parameters between the groups was made, using the one-way analysis of variance (ANOVA) by the statistical packages SPSS version 11.5. A value of *P*< 0.05 was considered a significant difference between groups.

## Results

***Phytochemical analysis***

Phytochemical analysis of the extract showed the presence of phenolic and flavonids content, 22.8±2.7 and 41.2±3.1 mg/g dry extract, respectively. Antioxidant activity by DPPH method showed 25.5±2.2 percent of scavenging activity. 

***Blood glucose concentrations***

**Figure 1:**
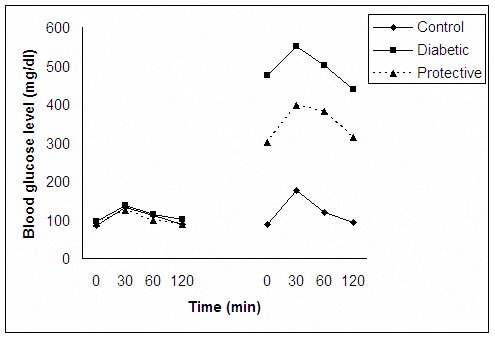
Glucose tolerance test (GTT) of the three groups of the experiment at the beginning (left) and the 5th week of the study (right).

The mean±SE of blood glucose concentrations, before injections of *U. dioica* extract and STZ, were 84.5±1.3, 95.3±4.5 and 91±4.2 mg/ dl in control, diabetic and protective groups, respectively. The mean±SE of blood glucose concentration level in control, diabetic and protective groups was 88.5±3.3, 475.2±39.6 and 301.7±80.1 mg/dl in the day 35. In the control group the mean±SE of blood glucose concentration did not show any changes. Statistically analysis showed that interaction between groups and days was significant (*P* =0.024). The glucose tolerance tests (GTT) of the three experiment groups at the beginning and the 5th week of the study are shown in Figure 1. As shown in the Figure, the GTT results of all rats were normal at the beginning of experiment. But at the end of the study, the GTT results showed prominent changes from the prior results of diabetic and protective groups (Figure 1).

***Histopathological findings***

**Figure 2:**
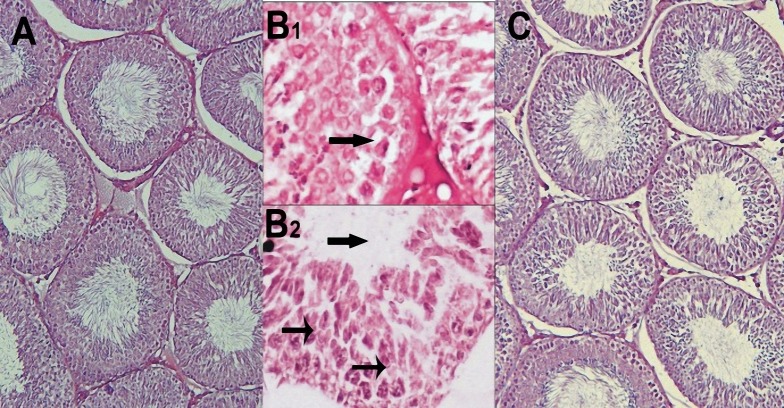
Histological architecture of testes. A- Protective group: no histopathological changes were seen in the seminiferous tubules. B _1, 2_ - Diabetic group: In B_1_ vacuolization and in B_2_ disintegration of the spermatogenic cells and decrease of spermatozoa in the seminiferous tubules are shown and C- Control group: the seminiferous tubules were shown natural structure (H&E staining, A and C: 100 ×, B _1, 2_: 400 ×) Morphometric results

In the control group, there was not any histopathological changes in seminiferous tubules and interstitial tissue. But in the diabetic group, disintegration of tubular cells, vacuolization of sertoli and spermatogonia cells were seen in most of seminiferous tubules. Also vasodilatation and congestion of capillaries were seen in interstitial tissue. In addition, spermatozoa were rarely seen in tubules in comparison with control group. Histopathological findings of the protective group were similar to control group but the thickness of tunica albuginea and fibrous tissue was increased (Figure 2). 

***Morphometric results***

The morphometric findings are depicted in Table 1. 

***Seminiferous tubular diameter (STD)***

The Mean±SE of external diameter of seminiferous tubules was 276.92±1.1, 233.04±1.22 and 254.31±1.39 micrometer in control, diabetic and protective groups, respectively. According to the data, STD in diabetic group was significantly lower than control group (*P*< 0.05). Also, STD increased in protective group in comparison with diabetic group (*P*< 0.05).

***Seminiferous epithelial height (SEH)***

The Mean±SE of seminiferous epithelial height was 76.59±0.54, 70.88±0.49 and 74.28±0.61 micrometer in control, diabetic and protective groups, respectively or in the given order. SEH was decreased in diabetic animals with the control group. This difference was considered significant (*P*< 0.05). On the other hand, SEH was increased in protective group in comparison with diabetic group (P< 0.05).

## Discussion

This study revealed that the administration of *U. dioica, *before induction of diabetes in animal model, has significant effect in preserving seminiferous tubules activity in diabetic rats. The decrease in morphometric indices such as STD in streptozotocin induced diabetic rats were reported by Altay (5) and other researchers (4, 9).

**Table 1:** Mean±SE of external seminiferous tubular diameter (STD) and seminiferous epithelial height (SEH) in control, diabetic and protective groups.

	Control (6)	Diabetic (6)	Protective (6)
STD (µm)	276.92±1.1	233.04±1.22 ^a^	254.31±1.39 ^ b^
SEH (µm)	76.59±0.54	70.88±0.49 ^a^	74.28±0.61 ^ b^

In our study, the morphometric indices such as STD and SEH decreased in diabetic rats but the preventive administration of *U. dioica *could increase STD and SEH in protective group. 

Also, histopathological alterations in STZ induced diabetic rats was reported by the researchers. In the present study, histopathological alterations in diabetic rats were observed. But these alterations in seminiferous tubules were not seen in the protective group which received *U. dioica *leaves extract before induction of diabetes. Some researchers reported that medicinal herbs had beneficial effects on reproduction in experimental diabetic rats (2,10).

Feng reported that *ligustrum* fruit extract diminished the damaging effect of experimental diabetes on spermatogenesis (11). Furthermore, Sangameswaran and Jayakar have shown that methanolic extract of *A. spinosus *L. stem has accelerated the process of spermatogenesis by increasing the sperm count and accessory sex organ weights (10).

Other reports indicated that herbal formulated drug named as MTEC consisting of aqueous-methanol *extract of Musa paradisiaca, Tamarindus indica, Eugenia jambolana and Coccinia indica* had a significant protective effect on testicular dysfunction in STZ induced diabetic rats (2).

The exact mechanism of the effect of* U. dioica* on seminiferous tubules is not clear. However, there are some possible mechanisms as following:

The preventive administration of *U. dioica* in the diabetic rats showed a significant recovery in fasting blood glucose level and regeneration of beta cells as proposed earlier by our previous study (12). The other possible mechanism is related to oxidative stress (OS). The oxidative stress is widely accepted as playing a direct key role in the pathogenesis of various diabetic complications (13,14). Shrilatha and Muralidhara reported that diabetes induction is associated with consistent OS in rat testis from first week onwards, which progresses with time. It is likely to contribute towards the development of testicular dysfunction as the oxidative impairments accompanied by compromised antioxidant defences and protein carbonyls in testes (13).

Furthermore, Mavi *et al* reported that *U. dioica* contains phenolic compounds, especially flavonoids (15). Flavonoids generally have antioxidant potential. Of course, for clearing the exact mechanism, further studies are needed.

## Conclusion

Regarding our results, we concluded that the administration of the hydroalcoholic extract of *U. dioica *leaves, before induction of diabetes, has a possible protective effect against histomorphometric alterations in seminiferous tubules of streptozotocin-induced diabetic rats. 
